# 

**Published:** 1896-03

**Authors:** 


### PERSONAL.

Dr. W. M. L. Coplin, formerly of Philadelphia, addressed the Farmers' Convention at Humboldt, Tenn., on the subject, "Shall the Live-stock Interests of Tennessee have Sanitary Protection?"

Veterinarian Jno. S. Meyer is taking a course in the medical college at St. Louis, Mo.

Prof. Liautard will remain abroad until some time in March. It is sincerely hoped by all that the health of Mrs. Liautard will be fully restored by the trip and change. Prof. Liautard recently announced to his extensive clientage his retirement from active practice.

Dr. F. A. Wall, of Pittsburg, is confined to a hospital, nursing injuries received in a street-car collision.

The home of Dr. Jas. A. Waugh, of Allegheny, has recently been gladdened by the birth of a son.

President Pearson has appointed the following members as a local committee of arrangements for the annual meeting of the Pennsylvania State Veterinary Medical Association at Philadelphia, March 3, 1896: Chairman, Dr. Jno. R. Hart, Dr. W. Horace Hoskins, and Dr. F. S. Allen.

The many friends of Prof. Jas. L. Robertson will be glad to learn of his gradual restoration to health, and wish for him that it may be speedy and complete.

E. E. Vaughn, V.S., late of Stamford, N. Y., has taken Dr. W. T. J. McLaughlin's office at 309 Barron Street, Jersey City, N. J.

MARRIED.—At Germantown, Philadelphia, February 12, 1896, by the Rev. James Lisk, Dr. Frank Kirk Nice, graduate of the American Veterinary College, to Miss Gertrude Louise Pullinger.

